# DID STRONGER STATE AGE DISCRIMINATION LAWS REDUCE THE ADVERSE IMPACTS OF COVID-19 ON LABOR OUTCOMES OF OLDER WORKERS?

**DOI:** 10.1093/geroni/igad104.1726

**Published:** 2023-12-21

**Authors:** Truc Bui

**Affiliations:** Tulane University, New Orleans, Louisiana, United States

## Abstract

Covid-19 caused tremendous impacts on the health and employment status of older workers, while current literature shows that age discrimination increased during recessions. In this context, I examine whether stronger state age discrimination laws reduced the adverse impacts of Covid-19 on the labor outcomes of older workers. State age discrimination laws are stronger than federal law because they protect workers in smaller firms or require firms to pay harsher penalties if violating the laws. I use these two classifications to identify the strength of state protections and employ the difference-in-difference-in-difference method. Using CPS and QWI data, I compare the labor outcomes of older and younger workers, in states with stronger and weaker laws, before and after the Covid-19 recession. There is very little evidence that stronger state laws protected older workers from the adverse impacts of the Covid-19 recession. I even found that stronger state protections were associated with more negative effects on older workers. Although stronger state laws were associated with higher hiring and separation rates right after the Covid-19 pandemic started, the increases in hiring rates were high enough to offset the growth in separation rates, and those effects were more pronounced among older men. The findings were consistent with the effectiveness of stronger state laws during the Great Recession, implying that severe labor market conditions weakened the effectiveness of stronger state age discrimination laws. The results also question the protection of discrimination laws on intersectional discrimination against age and gender.

